# Contrasting Phenotypes of Neutrophils During Asymptomatic Versus Symptomatic *Leishmania braziliensis* Infection

**DOI:** 10.1093/infdis/jiae317

**Published:** 2024-06-24

**Authors:** Jacilara A Conceição, Pedro P Carneiro, Andreza S Dórea, Walker N Oliveira, Aline C Muniz, Edgar M Carvalho, Mary E Wilson, Olívia Bacellar

**Affiliations:** Department of Internal Medicine, University of Iowa, Iowa City; Serviço de Imunologia, Hospital Universitário Prof. Edgard Santos, Universidade Federal da Bahia, Salvador, Brazil; Medical Service, Veterans’ Affairs Medical Center, Iowa City; Serviço de Imunologia, Hospital Universitário Prof. Edgard Santos, Universidade Federal da Bahia, Salvador, Brazil; Serviço de Imunologia, Hospital Universitário Prof. Edgard Santos, Universidade Federal da Bahia, Salvador, Brazil; Serviço de Imunologia, Hospital Universitário Prof. Edgard Santos, Universidade Federal da Bahia, Salvador, Brazil; Serviço de Imunologia, Hospital Universitário Prof. Edgard Santos, Universidade Federal da Bahia, Salvador, Brazil; Instituto Gonçalo Moniz, Fiocruz Bahia; Instituto Nacional de Ciência e Tecnologia de Doenças Tropicais, The National Council for Scientific and Technological Development/Ministry of Science and Technology, Salvador, Brazil; Department of Internal Medicine, University of Iowa, Iowa City; Medical Service, Veterans’ Affairs Medical Center, Iowa City; Department of Microbiology and Immunology, University of Iowa, Iowa City; Serviço de Imunologia, Hospital Universitário Prof. Edgard Santos, Universidade Federal da Bahia, Salvador, Brazil; Instituto Nacional de Ciência e Tecnologia de Doenças Tropicais, The National Council for Scientific and Technological Development/Ministry of Science and Technology, Salvador, Brazil

**Keywords:** neutrophil, PMNs, *Leishmania braziliensis*, subclinical infection, cutaneous leishmaniasis

## Abstract

**Background:**

The mechanisms that mediate immune protection in individuals with subclinical (SC) or asymptomatic infection with *Leishmania braziliensis* are largely unknown. Neutrophils (polymorphonuclear leukocytes [PMNs]) have been implicated in progressive symptomatic cutaneous leishmaniasis (CL), but their potential participation in maintenance of subclinical infection is unexplored. The aim of this study was to compare the phenotypic and functional profiles of PMNs in individuals with SC infection versus patients with symptomatic CL due to *L braziliensis*.

**Methods:**

Subjects were recruited in the endemic region of Corte de Pedra, Bahia, Brazil. Surface markers to define activation status were characterized by flow cytometry. Functional responses of PMNs including phagocytic capacity, production of oxidative species, and oxidative killing of intracellular parasites were studied in vitro.

**Results:**

PMNs from individuals with SC infection displayed a more activated phenotype and greater ability to control the infection than PMNs from patients with CL. In contrast, PMNs from patients with CL exhibited higher expression of HLA-DR and higher production of oxidative species than PMNs from subjects with SC infection.

**Conclusions:**

PMNs from individuals with SC infection can control the infection more efficiently than PMNs from patients with CL, despite the lower production of oxidants. Our observations suggest that *L braziliensis* may evade microbicidal mechanisms of PMNs from patients with CL, contributing to parasite dissemination and the establishment of disease.

## Teaser

Neutrophils from Brazilians who are subclinically infected with *Leishmania braziliensis* differ both phenotypically and functionally from neutrophils of individuals with symptomatic cutaneous leishmaniasis, suggesting they are primed for activation and better able to control infection.

Leishmaniasis refers to a chronic parasitic disease that assumes several clinical syndromes in humans. Cutaneous leishmaniasis (CL) is the most common form of leishmaniasis. Its immunopathogenesis involves a highly inflammatory adaptive immune response dominated by T-helper 1–type cytokines, which lacks appropriate regulation. This leads to tissue damage and consequently, ulcerated lesions [1–4]. *Leishmania braziliensis* can cause particularly severe CL, with a risk of dissemination to either mucosal or widespread cutaneous sites [5, 6]. Like other *Leishmania* spp, the spectrum of *L braziliensis* manifestations includes asymptomatic or subclinical (SC) infection, a state of great interest since it may reflect the ideal immune responses needed for protection. Subclinical *L braziliensis* infection is of particular interest because of the risk of developing destructive mucosal lesions months to years after cure of localized CL, or even when a primary lesion was not detected. Whether SC infection represents merely a period of latency before emergence of disseminated manifestations, or reflects the ideal balance between immunity and regulation, is not known.

Neutrophils (polymorphonuclear leukocytes [PMNs]) are the first cells to infiltrate the site of parasite inoculation in mouse models [ 7, 8], where they can exacerbate the immunopathogenesis of leishmaniasis through novel mechanisms. Murine models suggest that PMNs do not kill all intracellular *Leishmania* spp, but rather they can serve as reservoirs for viable promastigotes [7]. PMNs can promote macrophage infection through efferocytosis [9], and it is hypothesized that these contribute to suppression of macrophage microbicidal responses [10]. Infected neutrophils are also taken up by dendritic cells, suppressing their maturation to efficient antigen-presenting cells [11].

Studies of human PMNs incubated with *Leishmania* spp have revealed several responses to phagocytosis. These include up-regulation of surface markers suggesting activation, production of oxygen radicals, in vivo formation of neutrophil extracellular traps, and killing of intracellular parasites in vitro [12–14]. Despite activation, some *Leishmania* spp can hijack neutrophil microbicidal response and promote their own survival. For instance, *Leishmania major* evaded intracellular killing through impaired fusion between the parasitophorous vacuole and neutrophil granules [15].

PMNs seem to induce pathological responses during human leishmaniasis, especially in the more tissue-destructive disease forms. For example, the severe, damaging form of tegumentary disease, mucosal leishmaniasis, is accompanied by PMNs infiltrating lesions and expressing proteinases [16]. During visceral leishmaniasis, PMNs express high levels of arginase-1, an enzyme contributing to suppression of T-cell responses [17]. We previously detected a subset of PMNs expressing HLA-DR and other markers of antigen-presenting cells in both the circulation and lesion biopsies of patients with CL. HLA-DR^+^ PMNs displayed higher levels of activation markers and released more oxidants when compared to HLA-DR^−^ PMNs [18]. We also demonstrated that PMNs from patients with CL produce higher amounts of reactive oxidants than control uninfected persons, suggesting that PMNs may contribute to the proinflammatory environment [19]. Furthermore, this activated profile is altered to a basal phenotype after patient treatment and cure, reflecting the activity of disease [19].

The current study was based on the hypothesis that, whereas neutrophils contribute to the pathology observed in symptomatic forms of leishmaniasis, they are also critical for protection against *L braziliensis–*induced disease, and this is particularly evident during asymptomatic or SC infection. This would parallel our observation that monocytes and macrophages from individuals with *L braziliensis* SC infection exhibit greater ability to control the infection than cells from patients with active forms of clinical disease [20, 21]. We pursued this hypothesis by characterizing phenotypic and functional distinctions between PMNs from individuals with SC infection or active CL in the endemic region of Corte de Pedra, Bahia, Brazil [22]. Our results showed that PMNs from subclinical individuals displayed differences in their levels of activation and their functional capacities that could be associated with resistance to infection.

## MATERIALS AND METHODS

### Ethics Statement

This study was approved by the institutional review boards (IRBs) of the Federal University of Bahia (IRB number 51639) and the University of Iowa (protocol 200712745). All participants provided written informed consent prior to sample collection.

### Area of Study and Selection of Participants

This study was conducted in Corte de Pedra, Bahia, Brazil, an endemic area of *L braziliensis* transmission. Subjects were enrolled sequentially as they presented to the clinic at Corte de Pedra. Because individuals with SC infection must be identified by their skin test response in 2 sequential visits to their home 48 hours apart, they were selected from a prior 5-year cohort study of household contacts of patients with CL, begun in 2010 [21, 23]. Subjects were enrolled in this study between September 2015 and August 2016 during a return visit to their homes.

Individuals with SC were characterized by a positive *Leishmania* skin delayed-type hypersensitivity test (LST) and/or Quantiferon (blood cell production of interferon-γ specific to *Leishmania* antigen) and the absence of current or history of chronic cutaneous ulcers [21, 23]. Patients with CL were selected from subjects who came to the clinic in Corte de Pedra for evaluation of a skin ulcer. As part of the evaluation, an LST was applied and read 48–72 hours later. Patients with CL were identified by typical symptoms of CL, a positive LST, and confirmation of the parasite either by quantitative polymerase chain reaction (qPCR) for parasite sequences in DNA extracted from tissue samples, or microscopic identification of amastigotes by histopathologic examination of biopsies. Participants enrolled in this study included 19 individuals with SC infection and 33 patients with CL. Table 1 contains epidemiological and clinical characteristics of all individuals in this study for whom these data are available.

**Table 1.** Demographics and Lesion Sizes of Enrolled Subjects

Characteristic	Subclinical Infection		*P* Value (M-F)	Subclinical Infection	Cutaneous Leishmaniasis		*P* Value (M-F)	Cutaneous Leishmaniasis	*P* Value (SC-CL)
Sex (No. of subjects)	Male (9)	Female (7)		Overall (16)	Male (20)	Female (10)		Overall (30)	.49^a^
Age, y (mean [range])	24.80 (13.00–47.00)	22.00 (13.00–29.00)	.74	23.60 (13.00–47.00)	34.20 (18.00–61.00)	28.70 (17.00–53.00)	.18	32.40 (17.00–44.00)	.03
LST (length × width, mm)	20.25 × 17.00 mm	10.33 × 9.33 mm		16.00 × 13.71 mm	17.75 × 15.75 mm	14.80 × 13.60 mm		16.77 × 15.03 mm	
LST area (mm^2^)	270.23 mm^2^ ^b^	75.66 mm^2^ ^b^	.23	172.20 mm^2^	219.46 mm^2^	158.00 mm^2^	.30	197.86 mm^2^	.64
Lesion size (length × width, mm)	NA	NA		NA	18.92 × 14.23 mm	13.40 × 10.40 mm		17.39 × 13.17 mm	
Lesion area (mm^2^)	NA	NA		NA	211.35 mm^2^ ^c^	109.40 mm^2^ ^c^	.14	179.79 mm^2^	NA

Age, sex, and size of diagnostic LST are shown. For subjects with CL, the size of the lesion upon initial diagnosis, prior to therapy, is listed. LST and lesion size were the largest lesion dimension (length) × the dimension perpendicular to the length, in mm. Areas of LST test and lesions were determined by calculating the size of an oval of the same dimensions. All measurements are shown as the mean ± standard deviation in mm or mm^2^. Values are shown individually by sex and for both sexes (overall).

Abbreviations: CL, cutaneous leishmaniasis; F, female; LST, *Leishmania* skin test; M, male; NA, not applicable; SC, subclinical infection.

^a^Pearson χ^2^ test using values from the variables sex (male, female) and infection status (SC, CL).

^b^Values obtained from LST measurement of 4 males and 3 females among the group of individuals with SC infection. Two individuals displayed only positive LST while 5 displayed both positive LST and interferon-γ production specific to *Leishmania braziliensis* antigen.

^c^Values obtained from measurement of lesion size of 18 patients with CL (13 males and 5 females).

### Sample Collection and Human Neutrophil Isolation

Peripheral blood was obtained from the enrolled participants. In vitro infection experiments required PMN purification by density gradient centrifugation using Ficoll Paque Plus (GE Healthcare, United Kingdom). Peripheral blood mononuclear cell monolayer was collected, and red blood cells were removed by Dextran sedimentation (Pharmacosmos A/S, Denmark), leaving a population 95%–99% composed by PMNs. Purified PMNs were suspended in RPMI supplemented with 10% fetal bovine serum (FBS; Gibco, Thermo Fisher Scientific, Waltham, Massachusetts).

### Parasite Culture and Short-term Fluorescence Labeling

The parasite isolate was obtained from skin lesion biopsy of a patient with CL and characterized as *L braziliensis* (MHOM/BR/LTCP11245) by qPCR assay and isoenzyme electrophoretic mobility assay [24]. Parasites were cultivated in biphasic Novy-MacNeal-Nicolle medium [25], then cryopreserved in liquid nitrogen. For in vitro infection experiments, parasites were thawed, the culture was prepared in Schneider medium (LGC Biotecnologia, São Paulo, Brazil) supplemented with FBS, and incubated at 26°C until reach stationary phase. Stationary promastigotes were used at a 5:1 parasite:neutrophil ratio for infection experiments.

For some experiments, promastigotes were labeled with Cell Trace Far Red dichloro dimethyl acridin one succinimidyl ester (DDAO-SE) (Invitrogen, Thermo Fisher Scientific, Massachusetts) prior to addition to PMNs.

### In Vitro Infection of PMNs With *L braziliensis*

Stationary-phase *L braziliensis* promastigotes were prepared and added to neutrophil suspensions as described above. PMNs were incubated at 37°C, 5% CO_2_ for 90 or 180 minutes. Cytocentrifuged slides were prepared and stained with Wright-Giemsa. The percentage of infected cells and the number of internalized parasites was quantified by optical microscopy.

For oxidant detection assays, PMNs were exposed to DDAO-SE–labeled *L braziliensis* or stimulated with phorbol 12-myristate 13-acetate (PMA; Sigma-Aldrich, Merck, Germany) for 10 minutes in 10% FBS-supplemented RPMI. Unstimulated PMNs incubated under the same conditions were used as a negative control.

The influence of products of nicotinamide adenine dinucleotide phosphate (NADPH) oxidase on infection was investigated by adding 10 μM of diphenyleneiodonium chloride (DPI; Sigma-Aldrich, Merck, Germany) prior to parasite exposure. Cells were preincubated at 37°C, 5% CO_2_ for 10 minutes, then exposed to *L braziliensis* promastigotes for 3 hours. Untreated cells were used as negative control and submitted to the same condition. After 3 hours, cytocentrifuged slides were prepared, stained, and quantified by optical microscopy as described above.

### Neutrophil Phagocytic Capacity

Neutrophils’ phagocytic ability was evaluated by their capacity to internalize zymosan particles. Zymosan A (Sigma-Aldrich, Merck, Germany) was opsonized with fresh human serum for 10 minutes at 37°C 5% CO_2_, washed, and resuspended in RPMI. Ten micrograms of the suspension was added to PMNs and incubated at 37°C, 5% CO_2_ for 180 minutes. Cells in 100 μL of the suspension were applied to cytospin slides, fixed, and stained. The percentage of cells that internalized zymosan particles and the number of particles phagocytosed per neutrophil were quantified using an optical microscope as described above.

### Flow Cytometry for Peripheral Blood Neutrophil Surface Marker Detection

To assess the expression of surface markers on resting PMNs, 1 mL of whole blood was diluted in RPMI (1:5) (Gibco) and 100 μL of this dilution was stained directly ex vivo with the fluorochrome-conjugated monoclonal antibodies anti-CD15 fluorescein isothiocyanate, anti-CD66b PerCPCy5.5 (BD Pharmingen, San Diego, California), anti-CD62L PE-Cy7, anti-TLR2 PE, anti-TLR4 PE, and anti-HLA-DR PE (Biolegend, San Diego, California). BD FacsVerse was used for flow cytometry data acquisition and FlowJo software (TreeStar Inc, Ashland, Oregon) for data analyses.

### Detection of Oxidants Produced by Human PMNs

Two different probes were used to measure oxidant production by neutrophils. CM-H_2_DCFDA (2′,7′-dichlorodihydrofluorescein diacetate) is commonly used to detect total oxidant production. DAF-FM (4-amino-5-methylamino-2′,3′-difluorofluorescein) diacetate also detects oxidants, which can include nitric oxide (NO) derivatives if present. CM-H_2_DCFDA (1 µM, Invitrogen, Thermo Fisher Scientific) or DAF-FM diacetate (2 µM, Invitrogen) was added to neutrophil suspensions, and the cells were incubated at 37°, 5% CO_2_ for 15 minutes. PMNs were then stimulated with PMA or exposed to *L braziliensis* labeled with Cell Trace Far Red DDAO-SE (Invitrogen) at a 5:1 parasite ratio for 10 minutes. Cells were washed and stained with anti-CD15 PE (BD Biosciences, San Diego, California). Samples were acquired using BD FacsVerse and the data were analyzed using FlowJo software.

### Statistical Analyses

Statistical analysis was performed using GraphPad Prism software (GraphPad Software, San Diego, California). Assuming samples did not follow Gaussian distribution, analyses were performed using nonparametric tests. Wilcoxon paired or Friedman tests were used to compare results obtained with cells from the same individual exposed to 2 or more conditions, respectively. Mann-Whitney test was used to evaluate differences between individuals from different groups. *P* < .05 was considered statistically significant.

## RESULTS

### Differential Expression of Surface Markers on PMNs From Individuals With SC Infection Compared to PMNs From Patients With CL

We used flow cytometry to compare the basal status of circulating PMNs from individuals with subclinical infection compared to disease. Markers were chosen because the expression of CD62L is decreased and CD66b is increased during neutrophil activation. Furthermore, expression of TLR2 and TLR4 are upregulated during *Leishmania* infection [12].

**Figure 1.**
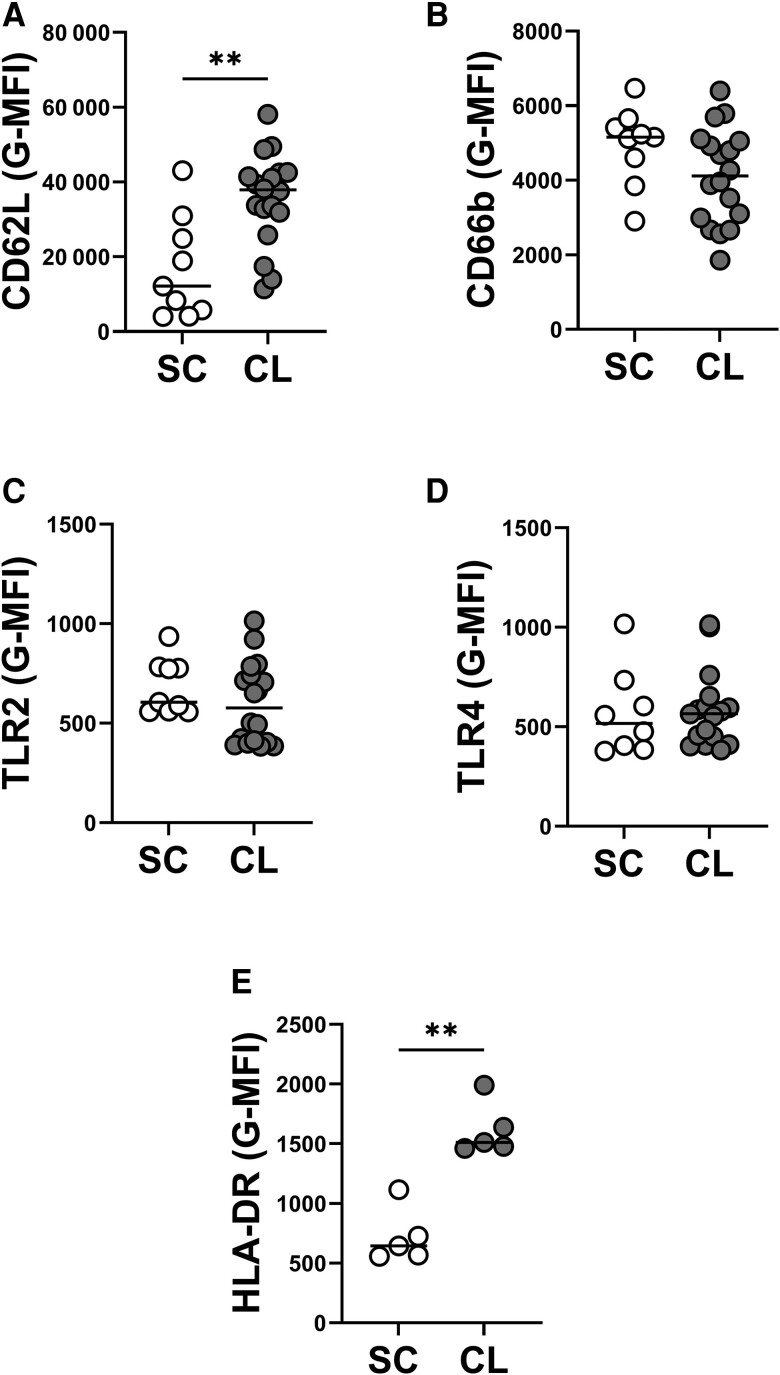
Surface markers expression on neutrophils from individuals with subclinical (SC) infection and patients with cutaneous leishmaniasis (CL). Whole blood was collected from individuals with SC infection (n = 9) or patients with CL (n = 18), then an aliquot was diluted with RPMI media and stained directly ex vivo with flow cytometry antibodies. Quantification of geometric mean fluorescence intensity (G-MFI) of CD15^+^ neutrophils expressing CD62L (*A*), CD66b (*B*), TLR2 (*C*), TLR4 (*D*), and HLA-DR (*E*) are shown above. Statistical analysis was performed using Mann-Whitney test (**P < .01).

Our data show a lower expression of CD62L, but no significant changes in CD66b on PMNs from individuals with SC infection compared to PMNs from CL patients (Figures 1*A* and 1*B*). There were no statistically significant differences in the expression of TLR2 and TLR4 when the groups were compared (Figures 1*C* and1*D*). HLA-DR is expressed by a subset of PMNs with features of antigen-presenting cells, called PMN-DC hybrids [26]. In the current study, PMNs from CL subjects had higher expression of HLA-DR than PMNs from SC individuals (Figure 1*E*).

### PMNs From Individuals With SC Infection Are Less Infected Than PMNs From Patients With CL After Exposure to *L braziliensis*

**Figure 2.**
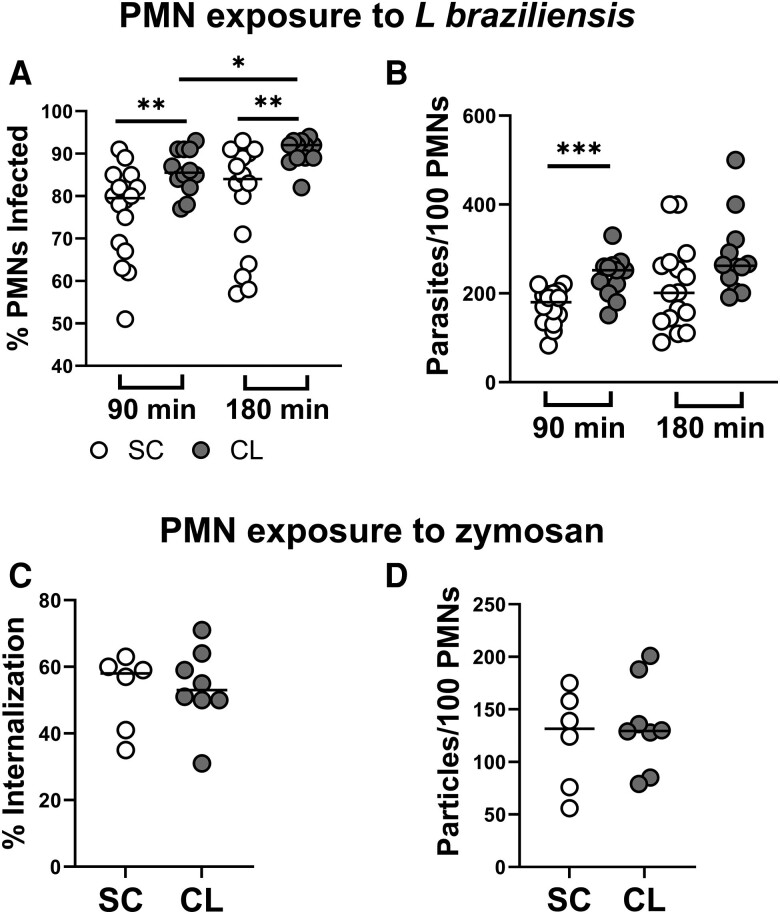
Neutrophils (polymorphonuclear leukocytes [PMNs]) from individuals with subclinical (SC) infection display greater ability to control *Leishmania braziliensis* in vitro infection than neutrophils from patients with cutaneous leishmaniasis (CL). Neutrophils were obtained from peripheral blood and exposed to *L braziliensis* for 90 or 180 min or zymosan for 180 min. Then, cytocentrifuged slides were prepared and stained with Giemsa. The frequency of cells with internalized parasite (*A*) or Zymosan particles (*C*) and the number of internalized parasites (*B*) or particles (*D*) per 100 PMNs were quantified by optical microscopy. Friedman test was applied for comparisons within the same group in different time points (*P < .05) and Mann-Whitney test, for comparisons between the groups (*P < .05; **P < .01).

Microscopy analysis of PMNs 90 or 180 minutes after exposure to *L braziliensis* revealed a lower frequency of infected cells (Figure 2*A*) and lower number of internalized parasites in PMNs from SC versus CL patients (Figure 2*B*).

The phagocytic capacity was further assessed by the capacity to internalize zymosan particles. PMNs were incubated with zymosan for 3 hours, and phagocytosis was assessed microscopically. Unlike parasite uptake, there were no statistically significant difference in the percentage of PMNs containing zymosan particles (Figure 2*C*) and the number of internalized particles/100 PMNs (Figure 2*D*) between PMNs from CL versus SC infection.

### Production of Oxidants by PMNs From SC Versus CL Subjects

**Figure 3.**
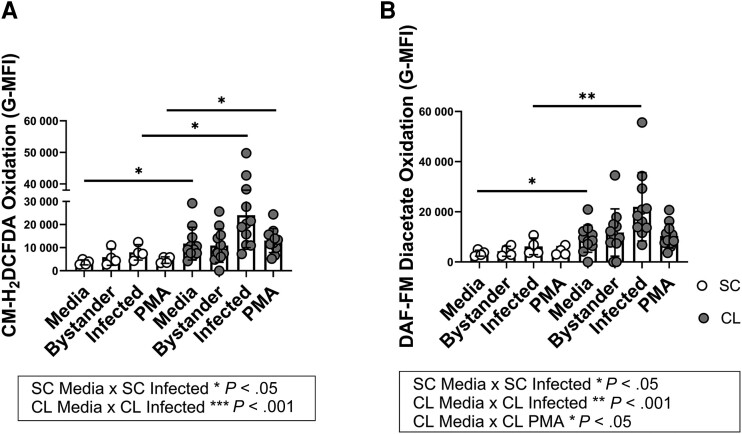
Production of oxidants by neutrophils from individuals with subclinical (SC) infection versus neutrophils from patients with cutaneous leishmaniasis (CL). Neutrophils were purified with Ficoll gradient and Dextran red blood cell sedimentation, stimulated with 2′,7′-dichlorodihydrofluorescein diacetate (CM-H_2_DCFDA; *A*) or 4-amino-5-methylamino-2′,3′-difluorofluorescein (DAF-FM) diacetate (*B*), then exposed to dichloro dimethyl acridin one succinimidyl ester–stained *Leishmania braziliensis* for 15 min and stained for anti-CD15. The oxidation of both probes in CD15^+^ neutrophils was measured by flow cytometry. Oxidant production by neutrophils toward different conditions was compared to unstimulated neutrophils in the same group using Friedman test (represented in boxes, *P < .05, **P < .01, ***P < .001) and to their respective condition in the other group using Mann-Whitney test (asterisks plotted on graphs, *P < .05, **P < .01). Abbreviations: G-MFI, geometric mean fluorescence intensity; PMA, phrobol 12-myristate 13-acetate.

Because 1 marker suggested a degree of activation in CL PMNs, PMNs were exposed to *L braziliensis* and the production of oxidants was assessed by flow cytometry using 2 different probes. PMNs exposed to PMA served as a positive control. The data showed that unstimulated PMNs from individuals with SC infection had minimal spontaneous oxidation of CM-H_2_DCFDA, in contrast to PMNs from patients with CL. Additionally, *L braziliensis*–infected or PMA-stimulated neutrophils from individuals with SC infection revealed significantly lower degree of CM-H_2_DCFDA oxidation when compared to cells in the same condition from patients with CL (Figure 3*A*).

Unstimulated or infected cells from the SC group displayed lower oxidation of DAF-FM diacetate when compared to cells from patients with CL (Figure 3*C*). Following infection, DAF-FM diacetate oxidation was enhanced in PMNs from both CL and SC groups when compared to the unstimulated cells (Figure 3*C*). CL PMNs also displayed higher NO production after stimulus with PMA (Figure 3*C*).

### NADPH Oxidase Activity Was Needed for Parasite Clearance by PMNs From Individuals With SC Infection and Patients With CL

**Figure 4.**
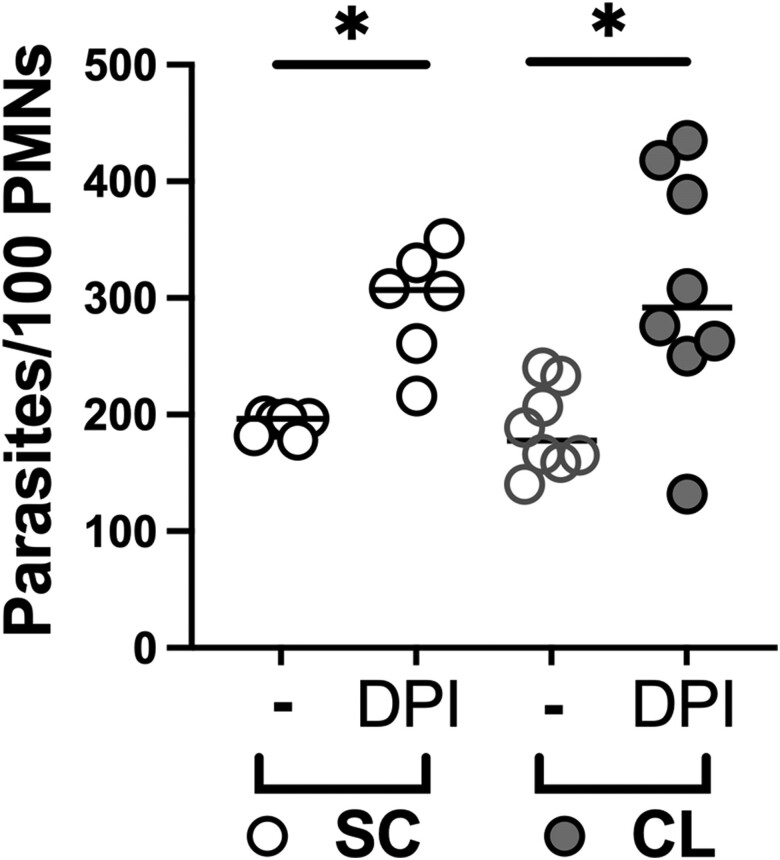
Nicotinamide adenine dinucleotide phosphate oxidase inhibition increases parasite burden in neutrophils (polymorphonuclear leukocytes [PMNs]) from both individuals with subclinical (SC) infection and patients with cutaneous leishmaniasis (CL). The number of internalized promastigotes of *Leishmania braziliensis* was compared in untreated neutrophils (−) or neutrophils treated with diphenyleneiodonium chloride (DPI) (Wilcoxon paired test, *P < .05) and between individuals with SC infection or CL (Mann-Whitney test).

Neutrophil was treated with DPI, an inhibitor of flavoenzymes including the NADPH oxidase, then exposed to *L braziliensis* to investigate the relevance of basal production of oxidants in infection control. The data showed an increase in parasite burden in DPI-treated PMNs from both the SC and CL groups after 3 hours of incubation with *L braziliensis* (Figure 4).

## DISCUSSION

Due to their short lifespan in circulation (6–8 hours) or tissues (up to 5–6 days), neutrophils are thought to be major determinants of the outcome of acute infectious diseases, but lesser so in chronic infections. CL due to *L braziliensis* is a chronic cutaneous ulcerative disease that occasionally disseminates to cause chronic disease in other skin sites. The influence of adaptive immune responses in symptomatic leishmaniasis has been widely documented. Some studies suggest that innate immune cells influence in the control of infection in asymptomatic individuals [20, 21]. Our prior studies have shown that the phenotype of PMNs differs between individuals with symptomatic CL compared to uninfected individuals [19]. Herein we investigated the hypothesis that, in contrast to symptomatic leishmaniasis, the innate neutrophil response is critical to protection against *L braziliensis–*induced disease during subclinical infection.

Working in an endemic area of northeast Brazil, our data revealed that PMNs from individuals with CL or with SC *L braziliensis* infection displayed distinct phenotypical and functional profiles. Neutrophils from individuals with SC infection exhibited lower expression of CD62L than those from patients with CL. The molecule CD62L or L-selectin is an integrin that participates in tethering to endothelium in preparation for transmigration into inflamed tissues. Although it is necessary for efficient neutrophil adhesion to endothelium, is shed enzymatically in response to adhesion and rolling on surfaces, but also in response to chemoattractants and chemokines. After tethering to an endothelial surface, CD62L is shed in response to adhesion, leading to increased velocity of rolling an neutrophil transmigration across the vascular endothelium [27–30]. Our observations might indicate that circulating neutrophils from individuals with SC infection are less likely to adhere to endothelial surfaces and thus less likely to migrate into infected tissues. It is important to note that decreased expression of CD62L occurs during neutrophil activation, but full activation would be accompanied by other functional and surface molecule changes such as decreased CD66b. Our finding could indicate that neutrophils from SC subjects are “primed” for rapid activation.

Previously, we identified an HLA-DR^+^ PMN population circulating in peripheral blood from patients with CL; this subpopulation was also detected in lesion biopsies from all evaluated patients, suggesting that those hybrid PMNs are more likely to migrate to the lesion site and could be involved in the inflammatory response associated with the pathology [18]. These were consistent with a subpopulation of PMNs described in 2013 by Matsushima and collaborators termed PMN-DC hybrids, which exhibit features of PMNs and dendritic cells, including MHC-II expression [26]. Our current findings suggest that these PMN-DC–like cell hybrids are a characteristic of patients with CL but not asymptomatic infection, indicating that they might contribute to the pathologic changes of CL.

Our group previously showed that monocytes from individuals with SC *L braziliensis* infection had lower phagocytosis but greater intracellular killing of the parasite [20, 21]. We made parallel observations in PMNs. There was a significantly lower uptake of parasites by PMNs from subjects with SC infection compared to disease. This observation was limited to phagocytosis of *L braziliensis* as opposed to inert zymosan particles.

The generation of reactive oxidants by PMNs from subjects with SC infection or CL was evaluated by the reduction of fluorescent dyes. The oxidation of CM-H_2_DCFDA is commonly used as an indicator of total oxidant production and was significantly induced in PMNs from both groups following exposure to *L braziliensis* when compared to unstimulated cells within the group. Increased oxidant production is consistent with reports demonstrating reactive oxygen species (ROS) production by PMNs from healthy donors following infection with *L braziliensis* or *Leishmania amazonensis* [12, 19, 31]. Comparison between the subject groups showed higher amounts of ROS produced both spontaneously and in response to PMA or parasite exposure by PMNs from patients with CL compared to SC subjects. This contrasts with our prior report showing that PMNs from CL patients produce more oxidants than healthy subjects after stimulation [19]. The reason for the lower oxidant capacity of PMNs from subjects with SC is not clear, although it could reflect ongoing or epigenetic changes induced by chronic low-level exposure to a latent pool of parasites.

A prior study showed that neutralization of TLR2 and TLR4 on monocytes from patients with CL caused them to be less susceptible to in vitro infection and generate lower amounts of oxidants, upon incubation with *L braziliensis* [32]. In a parallel study, neutrophils from healthy donors displayed reduced *L amazonensis* burdens after TLR2 neutralization in vivo [12]. In an experimental model of *L major* infection, it was also demonstrated that TLR7 is critical for efficient neutrophil microbicidal responses with ROS production and control of infection [33]. However, our observations directly ex vivo showed that TLR expression did not parallel either oxidant production or *L braziliensis* internalization in PMNs from patients with CL. Thus the role of TLRs in responses of neutrophils of patients with CL needs further clarification. The current study is the first to show that there are differences between entry of parasites into PMNs of infected humans without or with symptoms of CL, and that these differences are independent of the amount of surface TLR2 or TLR4.

We demonstrated that infection with *L braziliensis* triggered DAF-FM diacetate oxidation in PMNs from patients with CL. Comparison between the groups showed that either unstimulated or infected PMNs from individuals with SC infection generated lower amounts of oxidants than persons with symptomatic CL. Although DAF-FM diacetate has been cited as an NO indicator, we cannot affirm that its increased oxidation reflects NO production by neutrophils from CL in our study. It is important to acknowledge the limitations of probe usage on oxidant detection, since the performance can be affected by local O_2_ [34], or differences in peroxidases between cells, and the formation of artifacts cannot be disregarded [35]. Furthermore, it was documented that intraphagosomal nitration of fluorescein does not occur in human neutrophils [36]. Indeed, there are no reports showing NO production by human neutrophils using robust methods under optimal conditions. Thus, our findings indicate differences between the oxidative capacities of PMNs from subjects with CL or SC, but they do not implicate specific oxidant species.

Products of the NADPH oxidase were found to contribute to parasite control in PMNs from both groups. Thus, NADPH oxidase inhibition by the addition of DPI enhanced the numbers of intracellular parasites in PMNs from both SC and CL groups, although numbers of internalized parasites were still higher in subjects with CL. One hypothesis that could explain the inability of PMNs from patients with CL to control the intracellular parasite survival is that *L braziliensis* could evade exposure to oxidant species by the impaired fusion of intracellular granules with parasite-containing phagosomes. This would explain how infection persisted and amplified in the face of high spontaneous production of oxidant PMNs from patients with CL. This is consistent with a report showing that other species of *Leishmania* can evade PMN microbicidal activity by preventing granule fusion to the parasitophorous vacuole [15].

Considering the phenotypic and functional distinctions between SC and CL groups observed in our study, the evidence is consistent with a greater control of infection by PMNs from subjects with SC than patients with active disease. It is possible that *L braziliensis* can subvert the microbicidal mechanisms of PMNs from patients with localized cutaneous lesions, contributing to parasite survival and exacerbation of immunopathogenesis in CL. Whether this is due to differences in intracellular trafficking of the parasite in patients with SC infection or due to generalized defects in oxidative responses in patients with CL will require further investigations. Despite the fact that CL is a localized and a chronic infection, the data suggest that systemic responses of PMNs could contribute to or reflect the ability of the host to contain the parasite and remain free of disease.

## Notes

***Acknowledgments***. The authors thank Ednaldo Lago for his crucial collaboration in the endemic area of Corte de Pedra during the recruitment of individuals with subclinical infection and to Anderson Guimaraes for his much-appreciated contribution during the review of this manuscript.

***Author contributions*.** Conceptualization: J. A. C., E. M. C., O. B., and M. E. W. Data curation: J. A. C. and A. C. M. Formal analysis: J. A. C. Funding acquisition: O. B. and M. E. W. Investigation: J. A. C., P. P. C., A. S. D., W. N. O., and A. C. M. Methodology: J. A. C., O. B., E. M. C., and M. E. W. Project administration: O. B. and E. M. C. Supervision: O. B., E. M. C., and M. E. W. Validation: O. B. and M. E. W. Writing: J. A. C. and M. E. W.

***Financial support***. This research was supported by the Fundação de Amparo à Pesquisa do Estado da Bahia (Universal APP0059/2016 to O. B.); the National Institutes of Health (grant number AI088650 to E. M. C.); and the Department of Veterans' Affairs (Merit Review grant numbers I01 BX000536 and I01 BX001983 to M. E. W.). J. A. C. received a scholarship from the Coordination for the Improvement of Higher Education Personnel (Coordenação de Aperfeiçoamento de Pessoal de Nível Superior [CAPES]) and additional support from CAPES (Process: PDSE 88881.132700/2016-01) and the Brazilian Council for Scientific and Technological Development (Conselho Nacional de Desenvolvimento Científico e Tecnológico [CNPq], process: 205170/2018-8/PDE) to conduct research abroad during the development of this study. E. M. C. and O. B. are senior researchers at the CNPq.
